# The association between the retail price of manufactured cigarettes and bidis on current smoking status in India

**DOI:** 10.18332/tid/146904

**Published:** 2022-05-06

**Authors:** Radhika Nayak, Asha Kamath, Jinshuo Li, Muralidhar M. Kulkarni, Veena G. Kamath, Praveen Kumar, Ashwath Naik, Steve Parrott, Noreen D. Mdege

**Affiliations:** 1 Department of Community Medicine, Kasturba Medical College, Manipal Academy of Higher Education, Manipal, India; 2 Department of Data Science, Prasanna School of Public Health, Manipal Academy of Higher Education, Manipal, India; 3 Department of Health Sciences, University of York, York, United Kingdom; 4 Department of Commerce, Manipal Academy of Higher Education, Manipal, India

**Keywords:** tobacco use, GATS, price, current smoking

## Abstract

**INTRODUCTION:**

In India, the retail prices of bidis and cigarettes varied between the two Global Adult Tobacco Surveys (GATS) conducted in 2009–2010 and 2016–2017. The relationship between the retail price of smoked tobacco products and their use is unclear for India. Our study thus aimed to use available datasets to investigate the association between the retail price and current smoking status of bidis and cigarettes in India.

**METHODS:**

Current smoking status data for bidis and cigarettes were obtained from the two GATS rounds. The average state-level retail prices of bidis and cigarettes were obtained from India’s Consumer Price Index- Industrial Workers database. Descriptive statistics were used to describe current smoking status patterns. Generalized Linear Mixed Models were used to investigate the association between the retail prices and current smoking status of bidis and cigarettes.

**RESULTS:**

For cigarettes, an increase in the average retail price by one Indian Rupee was associated with a reduction in the odds of being a current smoker of 7% (OR=0.925; 95% CI: 0.918–0.932, p<0.001). For bidis, the association between the retail price and current smoking status was not statistically significant (OR=1.01; 95% CI: 1.00–1.02, p=0.082).

**CONCLUSIONS:**

Current increases in the retail prices of tobacco products in India seem to have an impact on the use of cigarettes but not bidis. This highlights the need for tobacco product tax increases that result in sufficient retail prices increase to make all tobacco products less affordable and reduce their use.

## INTRODUCTION

Tobacco use is one of the leading causes of death globally^1^. Eighty percent of mortality due to tobacco use is in low- and middle-income countries (LMICs)^2^. Tobacco smoking can result in serious health consequences such as tuberculosis, respiratory diseases, cardiovascular diseases and neoplasms^3^. Globally, India occupies the second position in both consumption and production of tobacco^3,4^. Bidis (tobacco hand-rolled, inexpensive, small and wrapped in dried tendu leaves) and cigarettes are two common tobacco smoking forms in India^5^. Compared to cigarettes, bidis are commonly used by people of low socioeconomic status due to their easy availability and lower cost^6,7^. Unfortunately, bidi smoking has been reported to be a stronger risk factor for cancer of the hypopharynx and supraglottis, as it appears to deliver some toxic components of tobacco smoke in greater amounts than conventional cigarettes^8^. The life expectancy of cigarette and bidi smokers is on average 6–10 years less than that of non-smokers^9^. According to India’s Global Adult Tobacco Surveys (GATS), the prevalence of tobacco use among adults in India has decreased from 34.6% to 28.6% between the periods 2009–2010 and 2016–2017, with 42.4% of men and 14.2% of women currently using tobacco^10^. Similarly, there has been a decline in the prevalence of current bidi smoking from 9.2% to 7.7% and of current cigarette smokers from 5.7% to 4.0% in the inter-survey period^10^. The consumption pattern of bidis and cigarettes varies in the 29 states and seven union territories in India due to diversity in culture, habits and economic status^3,5,11-13^. For instance, current prevalence of smoked forms of tobacco use in the southern state of Karnataka was 11.9% in 2009–2010 and 8.8% in 2016–2017, whereas in the northern territory of Delhi it was 17.4% and 11.3%, respectively^14,15^.

Tobacco product price is an important economic determinant of tobacco consumption^16^. Policies that increase the real consumer price (i.e. inflation adjusted) of tobacco products have been shown to reduce tobacco use, particularly if they reduce affordability of the products (i.e. the percentage of income required to buy specific units of a tobacco product)^17,18^. Taxation of tobacco products, for example, represents one of the most effective means of tobacco control: a 10% increase in tax could reduce cigarette smoking by 2%^19,20^. Tobacco taxes in India are complex in structure. During the study period, both central and state governments levied taxes on tobacco products. For example, for bidis and cigarettes, the central government imposed tax based on product characteristics such as stick length, presence of filter, whether machine or hand-made, and quantity. The state governments, on the other hand, had the authority to impose Value-added Tax (VAT) on tobacco products, resulting in varying tobacco product taxes and prices across states^21^. This has been cited as one of the reasons for the observed variations in tobacco use prevalence and consumption patterns across states^3,22,23^.

Tobacco companies work to limit the impact of taxes on tobacco product prices through market segmentation and setting lower prices for those consumers who are most price-sensitive, e.g. those of lower socioeconomic status. They achieve this, for example, through having different price tiers or point-of-sale price discounts offers. In order to offset these tobacco company strategies, there is an increased interest in non-tax policy approaches to raising tobacco product prices, for example minimum price laws that set a single floor price below which cigarettes cannot be sold^24-26^. Such strategies have been shown, through sales modelling studies, to potentially reduce smoking prevalence, with suggestions that the effects may be greater than achieved through taxation alone^24,25,27^. They also seem to have a greater relative impact on smokers in lower socioeconomic groups as tobacco product prices generally tend to be lower in more income-deprived neighborhoods, and hence could help reduce health inequalities^25,27,28^. Studies have demonstrated that higher cigarette prices have a negative effect on cigarette consumption^25,29^. A recent study concluded that higher bidi and cigarette prices can lower the probability of bidi or cigarette smoking onset in India^6^. However, the relationship between the retail prices of tobacco products and the prevalence of their use in India is unclear. We thus investigated the association between the retail prices and current smoking of cigarettes and bidis, adjusting for various sociodemographic factors and accounting for state-level variations.

## METHODS

### Data sources

Our analysis was based on data from the GATS in India and the Consumer Price Index for Industrial Workers (CPI-IW) database managed by the Labor Bureau Government of India (http://labourbureaucpi.gov.in/webform6.aspx)^30^.

India’s Ministry of Health & Family Welfare conducted two rounds of the GATS, one in 2009– 2010 (GATS-1), and the other in 2016–2017 (GATS-2)^14,15^. The GATS targets all Indian residents aged ≥15 years and living in their primary residence prior to the survey date. The GATS collects information on respondent’s demographic and socioeconomic characteristics, tobacco use (smoking and smokeless) and cessation, secondhand smoke exposure, tobacco related expenditures, media exposure to anti-tobacco information and tobacco advertisement, knowledge attitudes and perceptions towards tobacco use. There were 69296 and 74037 individual observations in GATS-1 and GATS-2, respectively. Data from these two GATS were combined for the analysis.

The CPI-IW database publishes state-level average monthly retail prices of tobacco products computed using data from selected industrially important centers based on brand name, filter/non-filter (for cigarettes), and number of sticks or units. The database contains information on a large number of local or subnational bidi brands, with one popular brand of national reach. The information on cigarette brands in the database were recorded for length shorter than 69 mm category for local or subnational brands. The database covers data starting from January 2006 to present. We assumed that any impact of change in retail price on individual-level smoking status would take at least a year to manifest itself^31^, hence we retrieved 2008 and 2015 retail prices, which were one year prior to GATS-1 and GATS-2 respectively.

### Dependent variables

For the two dependent variables, current bidi smoking status and current cigarette smoking status, we used responses to the following GATS question to categorize respondents as current bidi smoker/non-smoker and current cigarette smoker/non-smoker: ‘On average, how many of the following products do you currently smoke each day? Also, let me know if you smoke the product, but not every day’. Those who reported smoking one or more bidis each day, or smoking bidis but not every day, were considered as current bidi smokers; whilst those who indicated they did not smoke any bidis were current non-smokers (bidi). Similarly, those who reported smoking one or more manufactured cigarettes each day, or smoking manufactured cigarettes but not every day, were considered as current cigarette smokers; whilst those who indicated they did not smoke any manufactured cigarettes were current non-smokers (cigarette). The two variables were categorized independently of each other and did not consider dual users, i.e. smokers of both bidis and cigarettes, who constituted approximately 1.4% of our dataset.

### Independent variables

The average monthly retail prices, in Indian Rupees (INRs), for bidi and manufactured cigarettes were obtained from the CPI-IW database. As the pack sizes varied across products and states, the recorded retail prices were converted into prices of standard pack sizes in the Indian market: 25 sticks of bidis and 10 sticks of cigarettes. For each of the products, i.e. bidis and cigarettes, the retail price per standard pack for a state was estimated with a two-step average method: first the mean price of all products over the entire year for each center was calculated, then the average of the mean prices of all centers in a state was calculated as the retail price of a product for that state.

The following GATS sociodemographic variables were considered for the analysis based on empirical or theoretical literature reporting their association with current smoking status: age (as a continuous variable), residence (rural or urban), gender (female or male), level of education (no formal schooling, primary school, secondary school, higher secondary school or college and above), work status (government employee, non-government employee, self-employed, student, homemaker, retired or unemployed), smoking allowed in every room of house (yes or no), and wealth quintile (1=lower, 2=lower-middle, 3=middle, 4=middle-upper or 5=upper) based on modified Kuppuswamy socioeconomic scale 2020^32^. For our analysis, a few variables were recategorized from their original categories in GATS due to very few observations within each state. Specifically, for level of education, ‘Less than primary school completed’ and ‘primary school completed’ were recategorized as ‘primary school completed’, ‘less than secondary school completed’ and ‘secondary school completed’ were recategorized as ‘secondary school completed’, ‘college/university completed’ and ‘post graduate degree completed’ were recategorized as ‘College and above completed’; while ‘higher secondary school completed’ and ‘No formal schooling’ remained unchanged for the analysis. For work status, the GATS categories ‘daily wage/casual laborer’ and ‘self-employed’ were recategorized as ‘self-employed’; whilst categories ‘unemployed able to work’, and ‘unemployed unable to work’ were recategorized as ‘unemployed’; and categories ‘government employee’, ‘non-government employee’, ‘student’, ‘homemaker’ and ‘retired’ remained unchanged for the analysis. Values were considered missing when responses were blank, or the respondent refused to answer. Observations with missing values for any of the included variables were excluded from the analysis.

### Statistical analysis

The analysis was carried out using RStudio software version 3.6.1 (https://www.rstudio.com/). We conducted descriptive analysis, summarizing the average age of current smokers at the time of the survey, and the proportion of current smokers by the GATS derived sociodemographic variables, separately for bidis and manufactured cigarettes. To take into account the variation between states and GATS waves and predictors on both state-level and individual-level, we used Generalized Linear Mixed Models (GLMM) to analyze the association between retail price and current smoking status across the two GATS survey time points for bidis and cigarettes separately, controlling for sociodemographic variables, with states and GATS waves as random effect to account for clustering effect on state level and survey level^33^.

First, multilevel mixed-effects univariate logistic regression analyses were conducted to assess the empirical relationship between each independent variable and each dependent variable. The univariate analyses were used to select factors with p<0.2 for inclusion in the multivariate analyses^34,35^. For both current bidi smoking status and current cigarette smoking status, all independent variables had a p<0.2 in the univariate analyses, and were therefore included in multivariate analyses. For the multivariate analysis, odds ratios (ORs) and their 95% confidence intervals (CIs) were used as the measures of association, using a significance level of 0.05.

Hosmer-Lemeshow test and Akaike information criterion (AIC) were used to assess the model goodness-of-fit. Intraclass correlation coefficients (ICCs) were reported to attribute the variance accounted for by the states and the GATS survey period.

## RESULTS

For bidis, average retail price data were available for 23 of the 24 states included in the CPI-IW database. For these 23 states, we retrieved 82.3% observations from GATS-1 (57012/69296) and 81.0% observations from GATS-2 (59985/74037), constituting a total of 116997 observations after excluding observations with data missing on covariates. For cigarette smoking, average retail price data were available for all 24 states in the CPI-IW database. For these 24 states we retrieved 84.8% and 84.4% observations respectively from GATS-1 (58735/69296) and from GATS-2 (62476/74037), with a total of 121211 after excluding observations with data missing on covariates.

In GATS-1, the prevalence of bidi smoking and cigarette smoking in the analysis samples were 8.9% (5085/57012) and 6.1% (3603/58735), respectively; and in GATS-2 these were 7.8% (4695/59985) and 3.3% (2055/62476), respectively. The results of descriptive analysis are presented in Table 1. Table 2 shows the results of univariate analyses: all considered factors had a p<0.2 for both bidis and cigarettes and were included in the multivariate analysis.

**Table 1:** General characteristics of study population

*Characteristics*	*Bidis*				*Cigarettes*			
	*GATS-1*		*GATS-2*		*GATS-1*		*GATS-2*	
	*Overall n*	*Current bidi smokers n (%)*	*Overall n*	*Current bidi smokers n (%)*	*Overall n*	*Current cigarette smokers n (%)*	*Overall n*	*Current cigarette smokers n (%)*
**Age** (years), Mean ± SD	38.8 ± 14.9	45.2 ± 13.8	39.0 ± 15.7	45.8 ± 14.4	38.7 ± 14.9	40.4 ± 12.7	38.9 ± 15.7	39.4 ± 13.6
**Residence**								
Rural	32585	3664 (11.2)	37685	3681 (9.8)	33903	1811 (5.3)	39510	1111 (2.8)
Urban	24427	1421 (5.8)	22300	1014 (4.5)	24832	1792 (7.2)	22966	944 (4.1)
Gender								
Female	29135	454 (1.6)	32706	344 (1.1)	30037	120 (0.4)	34219	48 (0.1)
Male	27877	4631 (16.6)	27279	4351 (15.9)	28698	3483 (12.1)	28257	2007 (7.1)
**Education level**								
No formal schooling	15918	2019 (12.7)	15713	1731 (11.0)	16584	542 (3.3)	16506	281 (1.7)
Primary school completed	13410	1750 (13.0)	13068	1588 (12.2)	13712	931 (6.8)	13442	494 (3.7)
Secondary school completed	16162	999 (6.2)	17613	1096 (6.2)	16643	1210 (7.3)	18453	785 (4.3)
Higher secondary school	4930	183 (3.7)	6336	187 (3.0)	5073	379 (7.5)	6617	225 (3.4)
College and above completed	6458	105 (1.6)	7220	91 (1.3)	6589	538 (8.2)	7046	269 (3.6)
**Work status**								
Government employee	2850	184 (6.5)	2134	120 (5.6)	2966	391 (13.2)	2301	154 (6.7)
Non-government employee	11085	1466 (13.2)	17668	2329 (13.2)	11164	1174 (10.5)	17850	940 (5.3)
Self-employed	15728	2564 (16.3)	10546	1535 (14.6)	16151	1571 (9.7)	10969	711 (6.5)
Student	4151	16 (0.4)	4485	11 (0.2)	4388	99 (2.3)	4774	49 (1.0)
Home maker	19994	390 (2.0)	21540	268 (1.2)	20702	140 (0.7)	22848	65 (0.3)
Retired	1033	135 (13.1)	1387	136 (9.8)	1051	97 (9.2)	1436	53 (3.7)
Unemployed	2100	322 (15.3)	2204	295 (13.4)	2201	129 (5.9)	2274	83 (3.6)
**Wealth quintile**								
Lower	13124	1771 (13.5)	12194	1516 (12.4)	13317	481 (3.6)	12749	290 (2.3)
Lower-middle	10638	1288 (12.1)	13721	1494 (10.9)	10882	596 (5.5)	14085	446 (3.2)
Middle	10687	1020 (9.5)	10707	823 (7.7)	11246	800 (7.1)	11196	416 (3.7)
Middle-upper	11433	701 (6.1)	11478	593 (5.2)	11803	867 (7.3)	11990	448 (3.7)
Upper	11130	305 (2.7)	11885	269 (2.3)	11487	859 (7.5)	12456	455 (3.7)
**Smoking allowed in every room**								
No	12543	1141 (9.1)	11001	1265 (11.5)	13073	1119 (8.6)	12001	565 (4.7)
Yes	11520	2445 (21.2)	9226	1876 (20.3)	12051	1046 (8.7)	10058	533 (5.3)

**Table 2:** Association of sociodemographic and economic factors with current smoking status (bidis and cigarettes) in the study population, including two waves of GATS, from univariate analysis

*Factors*	*Current bidi smoking*			*Current cigarette smoking*		
	*Current smoker n (%)*	*OR (95% CI)*	*p*	*Current smoker n (%)*	*OR (95% CI)*	*p*
**Age** (years), Mean ± SD	45.5 ± 14.1	1.03 (1.028-1.031)	<0.001	40.1 ± 13.1	1.01 (1.004-1.007)	<0.001
**Residence**						
Rural	7345 (10.5)	1		2922 (4.0)	1	
Urban	2435 (5.2)	0.47 (0.45-0.49)	<0.001	2736 (5.7)	1.41 (1.33-1.49)	<0.001
**Gender**						
Female	798 (1.3)	1		168 (0.3)	1	
Male	8982 (16.3)	15.20 (14.12-16.36)	<0.001	5490 (9.6)	40.12 (34.45-46.73)	<0.001
**Education level**						
No formal schooling	3750 (11.9)	9.38 (8.13-10.81)	<0.001	823 (2.5)	0.41 (0.37-0.45)	<0.001
Primary school completed	3338 (12.6)	10.76 (9.32-12.41)	<0.001	1425 (5.2)	0.88 (0.81-0.96)	0.005
Secondary school completed	2095 (6.2)	4.82 (4.17-5.57)	<0.001	1995 (5.7)	0.98 (0.90-1.07)	0.632
Higher secondary school completed	370 (3.3)	2.32 (1.95-2.75)	<0.001	604 (5.2)	0.92 (0.82-1.02)	0.108
College and above completed	196 (1.4)	1		807 (5.8)	1	
**Work status**						
Government employee	304 (6.1)	1		545 (10.3)	1	
Non-government employee	3795 (13.2)	2.74 (2.43-3.09)	<0.001	2114 (7.3)	0.76 (0.69-0.84)	<0.001
Self-employed	4099 (15.6)	3.18 (2.82-3.59)	<0.001	2282 (8.4)	0.78 (0.71-0.86)	<0.001
Student	27 (0.3)	0.05 (0.03-0.07)	<0.001	148 (1.6)	0.15 (0.12-0.18)	<0.001
Home maker	658 (1.6)	0.25 (0.22-0.29)	<0.001	205 (0.5)	0.04 (0.04-0.05)	<0.001
Retired	271 (11.2)	2.22 (1.87-2.63)	<0.001	150 (6.0)	0.61 (0.50-0.73)	<0.001
Unemployed	617 (14.3)	2.91 (2.52-3.35)	<0.001	212 (4.7)	0.45 (0.38-0.53)	<0.001
**Wealth quintile**						
Lower	3287 (13.0)	6.23 (5.69-6.81)	<0.001	771 (3.0)	0.51 (0.47-0.56)	<0.001
Lower-middle	2782 (11.4)	5.79 (5.28-6.35)	<0.001	1042 (4.2)	0.76 (0.70-0.83)	<0.001
Middle	1843 (8.6)	4.15 (3.77-4.56)	<0.001	1216 (5.4)	0.96 (0.89-1.04)	0.360
Middle-upper	1294 (5.6)	2.50 (2.26-2.76)	<0.001	1315 (5.5)	0.99 (0.92-1.07)	0.823
Upper	574 (2.5)	1		1314 (5.5)	1	
**Smoking allowed in every room**						
No	2406 (10.2)	1		1684 (6.7)	1	
Yes	4321 (20.8)	2.28 (2.16-2.41)	<0.001	1579 (7.1)	1.07 (1.00-1.15)	0.061
**Average retail price of 25 sticks of bidis**	-	0.96 (0.95-0.97)	**<0.001**			
**Average retail price of ten cigarettes**				-	0.94 (0.94-0.95)	**<0.001**

In the multivariate analysis, the average retail price of cigarettes was statistically significantly associated with current smoking status for cigarettes. When the average retail price increases by one Indian Rupee, the odds of being a current smoker are reduced by 7% (OR=0.925; 95% CI: 0.918–0.932, p<0.001) (Table 3). An ICC of 23% and 11% was obtained, implying 23% of variation in current cigarette smoking status is attributed to GATS survey periods and 11% attributed to state variation. For bidis, the association between the retail price and current smoking status was not statistically significant (OR=1.01; 95% CI: 1.00–1.02, p=0.082). An ICC of 1% and 11%, indicated that only 1% of the variation in current smoking status is attributed to GATS survey period and 11% attributed to state variation.

**Table 3:** Association of sociodemographic and economic factors with current smoking status (bidis and cigarettes) in the study population inclusive of two waves of GATS, from multivariate analysis

*Factors*	*Current bidi smoking*		*Current cigarette smoking*	
	*AOR (95% CI)*	*p*	*AOR (95% CI)*	*p*
**Age**	1.03 (1.024–1.028)	<0.001	0.999 (0.997–1.001)	0.332
**Residence**				
Rural	1		1	
Urban	0.83 (0.78–0.88)	<0.001	1.33 (1.25–1.41)	<0.001
**Gender**				
Female	1		1	
Male	17.70 (15.92-19.69)	<0.001	39.86 (32.48-48.93)	<0.001
**Education level**				
No formal schooling	6.91 (5.87-8.13)	<0.001	0.94 (0.83-1.06)	0.329
Primary school completed	5.51 (4.70-6.47)	<0.001	1.12 (1.00-1.24)	0.048
Secondary school completed	3.21 (2.74-3.76)	<0.001	1.22 (1.11-1.34)	<0.001
Higher secondary school completed	2.13 (1.78-2.56)	<0.001	1.14 (1.01-1.28)	0.029
College and above completed	1		1	
**Work status**				
Government employee	1		1	
Non-government employee	1.15 (1.00-1.32)	0.047	0.90 (0.80-1.00)	0.049
Self-employed	1.02 (0.89-1.18)	0.733	0.81 (0.72-0.90)	0.0001
Student	0.08 (0.05-0.12)	<0.001	0.20 (0.17-0.25)	<0.001
Home maker	0.75 (0.63-0.90)	0.001	0.79 (0.64-0.98)	0.029
Retired	0.61 (0.50-0.74)	<0.001	0.53 (0.44-0.65)	<0.001
Unemployed	0.75 (0.64-0.89)	0.001	0.60 (0.50-0.72)	<0.001
**Wealth quintile**				
Lower	1.85 (1.65-2.07)	<0.001	0.41 (0.36-0.46)	<0.001
Lower-middle	2.22 (1.99-2.48)	<0.001	0.60 (0.54-0.66)	<0.001
Middle	1.88 (1.68-2.10)	<0.001	0.77 (0.70-0.85)	<0.001
Middle-upper	1.53 (1.37-1.71)	<0.001	0.87 (0.80-0.95)	0.002
Upper	1		1	
**Smoking allowed in every room**				
No	1		1	
Yes	6.02 (5.64-6.42)	<0.001	2.95 (2.76-3.15)	<0.001
**Average retail price of 25 sticks of bidis**	1.01 (1.00-1.02)	**0.082**	-	
**Average retail price of ten cigarettes**	-		0.925 (0.92-0.93)	<0.001

Older individuals were more likely to be bidi smokers, while the difference in age for cigarette smoking was not statistically significant. Those who were male, were more likely to be current smokers for both bidis and cigarettes compared to females. In addition, those living in a household where smoking was allowed in every room were more likely to be current cigarette and bidi smokers than those living in a household where smoking was not allowed in every room. Whilst those living in urban areas were less likely to be current bidi smokers than those living in the rural areas (OR=0.83; 95% CI=0.78– 0.88, p<0.001), they were more likely to be current cigarette smokers than those living in the rural areas. Compared to those who completed college or above education, people with lower education levels (primary, secondary and higher secondary schooling) were more likely to be smoking bidis. For cigarettes, while those in the middle levels of education were more likely to be smoking than those with college and above education, no difference was found between the lowest level of education category (no formal schooling) and the highest (college and above). Student, home maker, the unemployed and retired, were less likely to be current bidi and cigarette smokers compared to government employees. Self-employed people were less likely than government employees to be smoking cigarettes but did not differ in likelihood of smoking bidis. Those in the lower, lower-middle, middle and middle-upper wealth quintiles were more likely to be current bidi smokers, while those in the upper wealth quintile were more likely to be cigarette smokers.

## DISCUSSION

In our study, an increase in the average retail price per standard pack of commonly sold brands of manufactured cigarettes by one Indian Rupee was accompanied by a reduction in the odds of being a current smoker of 7%, taking clustering effect of states and GATS survey periods, and effects of other sociodemographic factors into consideration. For bidis, the association between the retail price and current smoking status was not statistically significant.

In India, tax levels for bidis are significantly lower than those for cigarettes and smokeless tobacco products^36^. In addition, the bidi industry has many small producers who take advantage of the tax concessions that are available for small producers^36,37^. Bidis are therefore cheaper, and tend to be more affordable at lower increments in tax/price when compared to cigarettes^36,38^. For example, in their projection of the affordability of cigarettes and bidis from 2017 to 2025, Rana et val.^39^ found that whilst the affordability for cigarettes decreased to -9.9% after a 100% increase in tax, that of bidi decreased to -8.61% only after a 200% increase in tax by the end of 2025.

Because they are cheaper, bidis are usually smoked by people of lower socioeconomic status, whilst cigarettes tend to be smoked by those of higher socioeconomic status^40^. This is consistent with our findings where those in the upper wealth quantile were less likely to be bidi smokers but more likely to be cigarette smokers when compared to those in the lower to middle-upper wealth quantiles. However, in the present study we could not capture the scenario of switching to cheaper products since we could not follow individual decisions with the secondary database. With regard to residence, we found that those in urban areas were more likely to be current smokers of manufactured cigarettes and less likely to be current smokers of bidis than those in the rural areas. Our observations with respect to education status revealed a notable reduction in the likelihood of cigarette smoking with increasing levels of education. In contrast, the education effect on bidi smoking, although existed, was not as prominent. However, the reduction in users of cigarettes was higher in each education category than users of bidis, between the two GATS surveys. Although consumers in India perceive bidis to be an inferior product relative to cigarettes, there is still a possibility that cigarette smokers switch to bidi smoking when they cannot afford to buy cigarettes because of high price^41,39^. Thus, the fact that current increases in the price of bidis do not seem to reduce the likelihood of bidi use by individuals has a potential to result in widening of health inequalities between the rich and the poor, the educated and uneducated, and rural versus urban populations. In addition, if cigarette smokers shift to bidi use as the cigarettes become more expensive, the change in smoking prevalence in the country will be negligible^39^.

In India, retail prices of tobacco products vary widely between states due to a number of reasons. For example, during the two GATS periods, there was a VAT system which in central and state taxes was imposed separately. Transportation costs from producing states to consuming states may result in price differences across states for the same brand^42^. Income disparities between states also influence price variation, as well as affordability among tobacco products across the states^21^. The percentage changes in retail price on bidis and cigarettes between the two GATS periods varied widely between states. For example, the price of 25 sticks of bidis was INR 4.60 in West Bengal and INR 7.6 in Tamil Nadu states during 2009–2010, but in 2016–2017 this was INR 8.40 and 21.50, respectively. Similarly, the price of 10 sticks of cigarettes was INR13.30 in Haryana state and INR 23.40 in Gujarat state during 2009–2010, but in 2016–2017 this was INR 69.00 and 51.80, respectively.^21^ In our study, these state-level and time period differences had a significant impact on the changes in current cigarette smoking but not for bidi smoking across states. Previous studies by Abdulkader et al.^43^ and Subramanian et al.^44^ on the tobacco consumption pattern in various regions in India also demonstrated that tobacco control activities vary across the regions and between different states, and this variation contributes to different patterns of change in prevalence of smoking.

Our study used retail prices which do not account for inflation or income growth. This was due to lack of data to estimate affordability (i.e. the percentage of income required to buy specific units of bidis or cigarettes), which adjusts for the consumer’s purchasing power, and is thereby considered an important indicator of the impact of tobacco-control fiscal policies^21^. Nevertheless, tobacco product retail prices are a major economic determinant of tobacco demand; and our study provides empirical evidence to underscore the fact that increasing prices without taking income growth into account might not lead to the desired effect of reduction in smoking prevalence^21,17^.

### Strengths and limitations

For the purpose of analysis, we defined and calculated a single standard unit price. In reality, there are a variety of products and brands available for both bidis and cigarettes with different sizes. It is possible that users of certain size or brand might be more sensitive to price change than those of the other, and our analyses would have missed this difference. As our sample was drawn from GATS, it was naturally limited by the sample selection criteria of the survey. For instance, we might have missed a migrant population due to the criterion of living in the address prior to the survey date. Since the GATS involves data of individuals aged ≥15 years, we could not draw any conclusion regarding those aged <15 years who might be more sensitive to price change. The data on our outcome of interest, current smoking status, was retrieved from an existing source of GATS where it is collected through self-reporting. There is a social desirability bias when self-reporting behaviors such as smoking, especially among females, which could lead to under-reporting and therefore estimation errors. Dual smokers of bidi and cigarettes were not considered for analysis because of differences in retail price of bidis and cigarettes. However, only about 1.4% of observations in our dataset were dual smokers of bidis and cigarettes. The interaction between the various background characteristics with the states could not be explored due to singularities in the model estimation. The CPI-IW database did not include data on retail prices of tobacco products from the following states which were therefore excluded from analysis: Jammu and Kashmir, Uttarakhand, Sikkim, Arunachal Pradesh, Nagaland, Manipur, Mizoram, and Meghalaya. Cigarettes are sold in length that varies from 55 to 85 mm, but data in majority of states are only available for cigarettes shorter than 69 mm. We also did not include smokeless tobacco in our analysis. These limitations have an impact on the generalizability of our findings.

To the best of our knowledge, our study is the first to assess the association between the retail price of manufactured cigarettes and bidis and current smoking status in India, taking into account state level variations to fit into India’s national context. This study was conducted using a large dataset from high-quality sources, which increases confidence in the validity of the results. The linking of two national representative surveys with the price of the tobacco products over the survey period, is to the best of our knowledge a novel approach. Future studies could explore the impact of the Goods and Services Tax (GST) implemented in 2017 on the use of the different tobacco products; as well as the impact of retail prices on the use of smokeless tobacco products, which are the predominant type of tobacco products used in India. This would facilitate policy making and strengthening of tobacco control across all tobacco products, which will result in an improvement in the health of the general population in India.

## CONCLUSIONS

Our study suggests that current increase in the retail prices of smoked tobacco products in India seem to have an impact on manufactured cigarette use but not bidi use. This highlights the need for tobacco product tax increases that are sufficient to make all tobacco products less affordable and reduce their use. This is particularly so for bidis, which have remained more affordable at lower increments in tax compared to cigarettes. In addition, eliminating the tax exemptions for small producers, which are often exploited by bidi producers, could reduce their affordability and use.

## Data Availability

Data sharing is not applicable to this article as no new data were created.
